# Predicting fall risk among older adults in Chinese communities with advanced machine learning techniques: a retrospective study

**DOI:** 10.3389/fpubh.2025.1628493

**Published:** 2025-09-01

**Authors:** Aihong Liu, Lingling Zhang, Debin Huang, Lianlian Qu

**Affiliations:** ^1^Department of Nursing, Union Hospital, Tongji Medical College, Huazhong University of Science and Technology, Wuhan, China; ^2^Department of Critical Care Medicine, First Affiliated Hospital of Guangxi Medical University, Guangxi Clinical Research Center for Critical Care Medicine, Nanning, China

**Keywords:** older adults, falls, machine learning, predictive model, SHAP algorithm

## Abstract

**Background:**

This study aims to develop a advanced machine learning model to predict the fall risk among community-dwelling elders. This study could present actionable advices for early prevention of fall risk.

**Methods:**

Between October and December 2022, 977 older adults from the Hannan District of Wuhan were recruited. Data was collected using structured questionnaires. The sample was randomly split into training (732 participants) and testing (245 participants) sets at a 3:1 ratio. The primary outcome was the occurrence of fall. Five machine learning models—Random Forest (RF), Gradient Boosted Decision Tree (GBDT), Light Gradient Boosting Machine (LGBM), Extreme Gradient Boosting (XGBoost), and Categorical Features Gradient Boosting (CatBoost)—were evaluated against a Logistic Regression (LR) model. Model performance was assessed using AUC, accuracy, precision, sensitivity, specificity, and F1 score.

**Results:**

Among the 977 older adults, 195 experienced falls (20.0%). ROC curve analysis showed AUC values of LR, RF, LGBM, GBDT, XGBoost, and CatBoost were, respectively, 0.8390, 0.8632, 0.8614, 0.8544, 0.8705, and 0.8719. CatBoost had the highest AUC, indicating the best predictive performance. SHapley Additive exPlanations (SHAP) analysis identified key features influencing the CatBoost model: history of falls, comorbidities, polypharmacy, sleep disorders, ADL, TUG results, frailty status, and use of assistive devices.

**Conclusion:**

The fall risk prediction model for community-dwelling older adults, developed with CatBoost, showed excellent performance and can aid in early clinical assessment and fall prevention.

## Introduction

The aging population is a global trend that has intensified concerns over the health of older adults. Among various health issues, falls have emerged as a major international public health concern (1). In China, the prevalence of falls among seniors is notably elevated, with approximately 19.3% of individuals aged 65 years and older experiencing at least one fall annually (2). As the population ages, a growing number of older adults will be exposed to this risk (3). Falls can result in numerous adverse outcomes, such as severe injuries, diminished mobility, and loss of independence. They may also elicit negative psychological effects, including anxiety, depression, and fear of falling, all of which can hinder physical recovery (4, 5). Moreover, the economic burden associated with falls is considerable and continues to escalate globally. In 2015, medical expenses related to falls in the United States surpassed $50 billion (6). In China, it is estimated that 26 million older adults experience falls annually, leading to approximately 5 billion yuan in direct medical costs and 60–80 billion yuan in social costs (2). Consequently, identifying the risk factors for falls among community-dwelling older adults is essential for the early detection of at-risk populations and the formulation of effective prevention strategies.

Research has demonstrated that employing appropriate assessment tools to evaluate fall risk in older adults can facilitate the formulation of targeted interventions aimed at reducing incidents, thereby enhancing survival rates and quality of life while alleviating healthcare burdens (7, 8). Current predictive models for fall risk among community-dwelling older adults predominantly rely on traditional logistic regression (LR) methods. These models typically incorporate statistically significant variables identified via univariate and multivariate analyses but may inadvertently omit other clinically relevant factors, leading to a potential mismatch between predicted outcomes and actual fall occurrences. Machine learning, a cornerstone technology of artificial intelligence, excels in handling nonlinear relationships among complex variables, enabling the development of more robust risk prediction models (9). It has been extensively applied across various domains within medicine. Unlike traditional methods, machine learning algorithms do not necessitate prior assumptions about correlations between variables; instead, they analyze all available data to uncover patterns and learn from intricate datasets to identify potential predictive features (10).

Existing studies have emphasized the importance of incorporating electronic medical record (EMR) data, which is frequently updated and inherently comprehensive, thereby significantly improving the accuracy of fall risk prediction. However, the limited adoption of EMR systems in community settings (non-clinical environments) in China constrains their practical applicability. To address this gap, we collected data independently and employed advanced machine learning methods.

Given this context, this study utilizes multiple machine learning algorithms—namely LR, Random Forest (RF), LightGBM (LGBM), Categorical Features Gradient Boosting (CatBoost), Gradient Boosting Decision Trees (GBDT), and Extreme Gradient Boosting (XGBoost)—to develop a predictive model for fall risk among community-dwelling older adults. The study aims to assess and compare the performance of these models, identify the most effective one, and investigate the factors associated with falls, thereby providing a theoretical foundation for interventions designed to prevent falls in this population.

## Subjects and methods

### Study participants

This retrospective study was conducted between October and November 2022, employing convenience sampling to recruit older adults from four communities in the Hannan District of Wuhan. Inclusion criteria were as follows: (1) age ≥ 60 years; (2) residence duration ≥ 6 months; (3) ability to comprehend instructions and communicate effectively; (4) provision of informed consent to participate. Exclusion criteria included: (1) bedridden individuals; (2) individuals with paralysis or epilepsy; (3) individuals diagnosed with mental disorders; (4) Individuals who are unable to cooperate with on-site testing. A total of 25 potential risk factors were identified. Based on a sampling ratio of five times the number of independent variables, a necessary sample size of 125 was calculated. Considering the previously reported fall incidence rate of 18.8% among community-dwelling older adults in China, a minimum sample size of 664 was determined. Ultimately, 996 older adults were surveyed, and after data cleaning to exclude incomplete or anomalous entries, a valid sample of 977 participants was obtained. The dataset was randomized using Python and divided into training and testing sets at a ratio of 3:1. This study was approved by the Ethics Committee of Union Hospital, Tongji Medical College, Huazhong University of Science and Technology (approval number: 0312). Informed consent was obtained from all the participants.

### Study tools

### Determining influencing factors

The research team systematically examined relevant studies on fall risk among community-dwelling older adults, both domestically and internationally. Shao et al. (11) conducted a meta-analysis and found that risk factors such as a history of falls, impaired ADL performance, insomnia, and depression are strongly associated with falls. The 2022 guidelines recommend conducting a multifactorial fall risk assessment, including ADL assessment, cognitive screening, balance testing, disease, hearing, vision, frailty, geriatric depression, and other areas (12). Following in-depth discussions, we identified 25 risk factors associated with falls in this population. These factors include gender, age, marital status, educational attainment, living arrangements, the presence of multiple chronic conditions, polypharmacy, a history of falls within the past year, smoking and alcohol consumption habits, and the use of assistive devices. Subsequently, data were collected via face-to-face surveys and functional assessments to obtain pertinent information.

### Instrumental activities of daily living scale

Developed by Lawton et al. in 1969, this scale assesses the capability for independent living across eight domains: telephone use, shopping, meal preparation, household chore management, laundry, transportation use, medication management, and financial management. Scores range from 0 to 8, with scores below 7 indicating functional limitations (13).

### Frailty assessment: fried frailty phenotype

This assessment tool comprises five criteria: slow walking speed, weight loss, fatigue, weakened grip strength, and reduced physical activity. Each criterion is scored as 1 for “meets the criterion” and 0 for “does not meet the criterion.” The total score indicates the frailty status as follows: 0 (no frailty), 1–2 (pre-frailty), and ≥ 3 (frailty) (14).

### Physical function tests

The Four-Stage Balance Test (4-SBT) evaluates static balance through four progressively challenging tasks: standing with feet side by side, semi-tandem standing, tandem standing, and single-leg standing. A score of 4, achieved by completing all tasks successfully, indicates adequate static balance (15). The Timed Up and Go Test (TUGT) assesses mobility and balance by measuring the time it takes for a participant to stand up from a seated position, walk 3 m, turn around, return to the chair, and sit down; a completion time of ≥ 12.3 s suggests an increased fall risk (16). The Five Times Chair Stand Test evaluates lower body strength, with a completion time of ≤ 11.1 s indicating excellent leg strength (17).

### Pittsburgh sleep quality index (PSQI)

The PSQI evaluates sleep quality across seven domains: subjective sleep quality, sleep latency, sleep duration, habitual sleep efficiency, sleep disturbances, use of sleeping medications, and daytime dysfunction. A total score > 7 indicates the presence of significant sleep disturbances (18).

### Geriatric depression scale (GDS-15)

This scale is utilized to assess depressive symptoms experienced during the past week. It comprises five items, with total scores ranging from 0 to 15; higher scores signify more severe depressive symptoms (19).

### Mini-mental state examination (MMSE)

The MMSE evaluates cognitive function across several dimensions, including orientation, memory, attention, and calculation, with total scores ranging from 0 to 30 (20).

### Data collection and entry

Two research team members, trained in comprehensive geriatric assessment, conducted the surveys, ensuring their ability to effectively administer the various measurement tools. With the cooperation of community staff and based on a fundamental understanding of the community, participants were gathered at community health service centers to complete on-site surveys. To enhance participant compliance, the research team provided each participant with a small incentive. Informed consent was obtained from both participants and their families, with detailed explanations provided regarding the study’s purpose, significance, and procedures. Baseline data were collected through face-to-face surveys and physical function tests, utilizing standardized scripts to ensure clarity and consistency. For individuals with low literacy levels or difficulties in understanding the questions, the enumerator will assume responsibility for administering the questionnaire, providing explanations, and completing the form on their behalf using language that is easily understood.

### Statistical analysis

Normally distributed data are presented as mean ± standard deviation, with independent samples *t*-tests used for group comparisons. Categorical data are reported as percentages and analyzed using chi-square tests. Non-normally distributed data are expressed as median (interquartile range, IQR) and compared using the Mann–Whitney U test. Statistical significance was set at *p* < 0.05. The machine learning models—LR, RF, LGBM, GBDT, XGBoost, and CatBoost—were evaluated alongside a traditional LR model. Model performance was assessed using metrics such as accuracy, sensitivity, specificity, F1 score, and the area under the receiver operating characteristic curve (AUC).

## Results

### Comparison of baseline data between fall and non-fall groups

**Figure 1:**
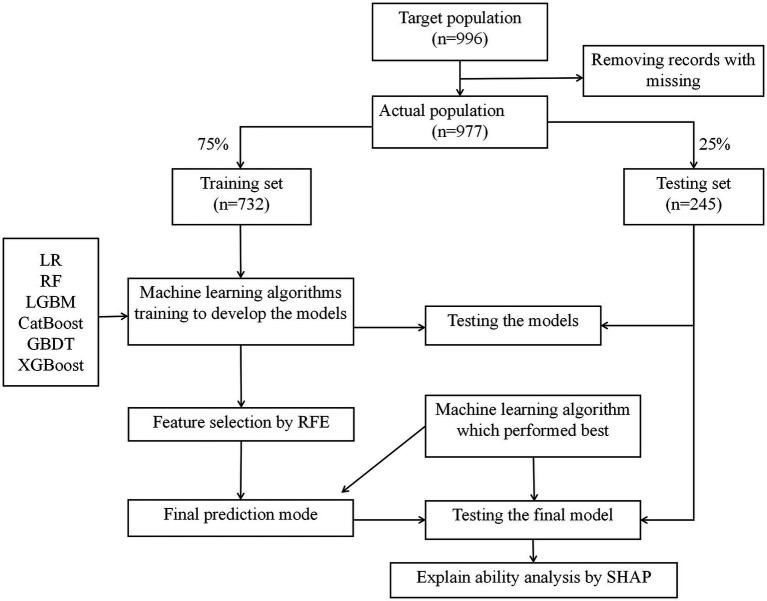
Consort flow diagram.

The study included 977 older adults aged 60–93 years, comprising 534 females (54%) and 443 males (46%). A total of 195 participants (20%) reported experiencing falls (Figure 1). Significant differences were observed between the fall and non-fall groups in terms of marital status, age, comorbidities, educational attainment, living alone, use of assistive devices, history of falls, hearing impairment, vision impairment, alcohol consumption, sleep disturbances, polypharmacy, activities of daily living (ADL), frailty status, and various physical assessments (*p* < 0.05). Conversely, no significant differences were found regarding gender, household income, smoking status, body mass index (BMI), or Mini Nutritional Assessment Short-Form (MNA-SF) scores (*p* > 0.05). For detailed information, refer to Tables 1, 2.

**Table 1:** Baseline characteristics of community-dwelling older adults.

Parameters	Total (*n* = 977)	No falls (*n* = 782)	Falls (*n* = 195)
Gender			
Male	443 (45.34%)	354 (45.27%)	89 (45.64%)
Female	534 (54.66%)	428 (54.73%)	106 (54.36%)
Age	71.0 (67.0, 75.0)	71.0 (67.0, 74.0)	74.0 (69.0, 80.0)
Marital status			
Married	827 (84.65%)	673 (86.06%)	154 (78.97%)
Divorce or widow	150 (15.35%)	109 (13.94%)	41 (21.03%)
Income			
< ¥3,000	68 (6.96%)	53 (6.78%)	15 (7.69%)
¥3,000–5,000	659 (67.45%)	525 (67.14%)	134 (68.72%)
>¥5,000	250 (25.59%)	204 (26.09%)	46 (23.59%)
Comorbidity			
No	537 (54.96%)	492 (62.92%)	45 (23.08%)
Yes	440 (45.04%)	290 (37.08%)	150 (76.92%)
Education			
Primary school and below	109 (11.16%)	63 (8.06%)	46 (23.59%)
Junior high school	475 (48.62%)	384 (49.1%)	91 (46.67%)
High school	321 (32.86%)	282 (36.06%)	39 (20.0%)
Universities	72 (7.37%)	53 (6.78%)	19 (9.74%)
Living alone			
No	792 (81.06%)	650 (83.12%)	142 (72.82%)
Yes	185 (18.94%)	132 (16.88%)	53 (27.18%)
Assistive devices			
No	905 (92.63%)	760 (97.19%)	145 (74.36%)
Yes	72 (7.37%)	22 (2.81%)	50 (25.64%)
Falls history			
No	781 (79.94%)	684 (87.47%)	97 (49.74%)
Yes	196 (20.06%)	98 (12.53%)	98 (50.26%)
Hearing impairment			
No	877 (89.76%)	721 (92.2%)	156 (80.0%)
Yes	100 (10.24%)	61 (7.8%)	39 (20.0%)
Vision impairment			
No	828 (84.75%)	687 (87.85%)	141 (72.31%)
Yes	149 (15.25%)	95 (12.15%)	54 (27.69%)
Smoking			
No	897 (91.81%)	723 (92.46%)	174 (89.23%)
Yes	80 (8.19%)	59 (7.54%)	21 (10.77%)
Drinking			
No	900 (92.12%)	741 (94.76%)	159 (81.54%)
Yes	77 (7.88%)	41 (5.24%)	36 (18.46%)
Sleep disorder			
No	849 (86.9%)	713 (91.18%)	136 (69.74%)
Yes	128 (13.1%)	69 (8.82%)	59 (30.26%)
Polypharmacy			
No	664 (67.96%)	600 (76.73%)	64 (32.82%)
Yes	313 (32.04%)	182 (23.27%)	131 (67.18%)
ADL			
<14	751 (76.87%)	633 (80.95%)	118 (60.51%)
14–21	182 (18.63%)	133 (17.01%)	49 (25.13%)
>21	44 (4.5%)	16 (2.05%)	28 (14.36%)
MNA-SF			
No	714 (73.08%)	575 (73.53%)	139 (71.28%)
Yes	263 (26.92%)	207 (26.47%)	56 (28.72%)
Frailty			
No	750 (76.77%)	652 (83.38%)	98 (50.26%)
Yes	227 (23.23%)	130 (16.62%)	97 (49.74%)
TUGT			
No	706 (72.26%)	615 (78.64%)	91 (46.67%)
Yes	271 (27.74%)	167 (21.36%)	104 (53.33%)
4-SBT			
No	739 (75.64%)	626 (80.05%)	113 (57.95%)
Yes	238 (24.36%)	156 (19.95%)	82 (42.05%)
FTSST			
No	748 (76.56%)	640 (81.84%)	108 (55.38%)
Yes	229 (23.44%)	142 (18.16%)	87 (44.62%)
MMSE			
No	760 (77.79%)	623 (79.67%)	137 (70.26%)
Yes	217 (22.21%)	159 (20.33%)	58 (29.74%)
GDS-15			
No	827 (84.65%)	674 (86.19%)	153 (78.46%)
Yes	150 (15.35%)	108 (13.81%)	42 (21.54%)
BMI	22.95 ± 2.79	23.17 ± 2.76	22.05 ± 2.76

ADL, activity of daily living; MNA-SF, mini nutritional assessment short-form; TUGT, timed up and go test; 4-SBT, 4-stage balance test; FTSST, five-times-sit-to-stand test; MMSE, mini-mental state examination; GDS-15, geriatric depression scale; BMI, body mass index.

**Table 2:** Comparison of general characteristics between the training and testing datasets.

Parameter	Training set (*n* = 732)	Testing set (*n* = 245)	*p*-value
Gender			
Male	335 (45.77%)	108 (44.08%)	0.701
Female	397 (54.23%)	137 (55.92%)	
Age	71.0 (67.0, 75.25)	71.0 (67.0, 74.0)	0.450
Marital status			
Married	615 (84.02%)	212 (86.53%)	0.399
Divorce or widow	117 (15.98%)	33 (13.47%)	
Income			
< ¥3,000	45 (6.15%)	23 (9.39%)	0.226
¥3,000–5,000	498 (68.03%)	161 (65.71%)	
>¥5,000	189 (25.82%)	61 (24.9%)	
Comorbidity			
No	414 (56.56%)	123 (50.2%)	0.098
Yes	318 (43.44%)	122 (49.8%)	
Education			
Primary school and below	83 (11.34%)	26 (10.61%)	0.441
Junior high school	345 (47.13%)	130 (53.06%)	
High school	249 (34.02%)	72 (29.39%)	
Universities	55 (7.51%)	17 (6.94%)	
Living alone			
No	601 (82.1%)	191 (77.96%)	0.181
Yes	131 (17.9%)	54 (22.04%)	
Assistive devices			
No	680 (92.9%)	225 (91.84%)	0.683
Yes	52 (7.1%)	20 (8.16%)	
Falls history			
No	589 (80.46%)	192 (78.37%)	0.537
Yes	143 (19.54%)	53 (21.63%)	
Hearing impairment			
No	658 (89.89%)	219 (89.39%)	0.918
Yes	74 (10.11%)	26 (10.61%)	
Vision impairment			
No	619 (84.56%)	209 (85.31%)	0.859
Yes	113 (15.44%)	36 (14.69%)	
Smoking			
No	676 (92.35%)	221 (90.2%)	0.355
Yes	56 (7.65%)	24 (9.8%)	
Drinking			
No	676 (92.35%)	224 (91.43%)	0.744
Yes	56 (7.65%)	21 (8.57%)	
Sleep disorder			
No	634 (86.61%)	215 (87.76%)	0.727
Yes	98 (13.39%)	30 (12.24%)	
Polypharmacy			
No	498 (68.03%)	166 (67.76%)	0.999
Yes	234 (31.97%)	79 (32.24%)	
ADL			
<14	564 (77.05%)	187 (76.33%)	0.937
14–21	136 (18.58%)	46 (18.78%)	
>21	32 (4.37%)	12 (4.9%)	
MNA-SF			
No	539 (73.63%)	175 (71.43%)	0.555
Yes	193 (26.37%)	70 (28.57%)	
Frailty			
No	555 (75.82%)	195 (79.59%)	0.262
Yes	177 (24.18%)	50 (20.41%)	
TUGT			
No	526 (71.86%)	180 (73.47%)	0.685
Yes	206 (28.14%)	65 (26.53%)	
4-SBT			
No	555 (75.82%)	184 (75.1%)	0.888
Yes	177 (24.18%)	61 (24.9%)	
FTSST			
No	553 (75.55%)	195 (79.59%)	0.228
Yes	179 (24.45%)	50 (20.41%)	
MMSE			
No	559 (76.37%)	201 (82.04%)	0.078
Yes	173 (23.63%)	44 (17.96%)	
GDS-15			
No	615 (84.02%)	212 (86.53%)	0.399
Yes	117 (15.98%)	33 (13.47%)	
BMI	22.86 (21.04, 24.7)	23.04 (21.08, 25.3)	0.374

ADL, activity of daily living; MNA-SF, mini nutritional assessment short-form; TUGT, timed up and go test; 4-SBT, 4-stage balance test; FTSST, five-times-sit-to-stand test; MMSE, mini-mental state examination; GDS-15, geriatric depression scale; BMI, body mass index.

### Fall risk prediction modeling for community-dwelling older adults

All relevant factors were incorporated into the modeling process, utilizing six machine learning algorithms: LR, RF, LGBM, CatBoost, GBDT, and XGBoost. Hyperparameter optimization was conducted using grid search to determine the optimal key parameters for the six algorithms. The selected optimal parameters for CatBoost are as follows: learning_rate = 0.01, depth = 4, iterations = 1,000, and early_stopping_rounds = 800, which contributed to identifying the most effective parameter configuration for the model. A consort flow diagram is presented in Figure 1 on p. 30. The results demonstrated that the CatBoost algorithm achieved the highest predictive performance, with an AUC of 0.8820 on the training set and 0.8719 on the testing set.

**Figure 2:**
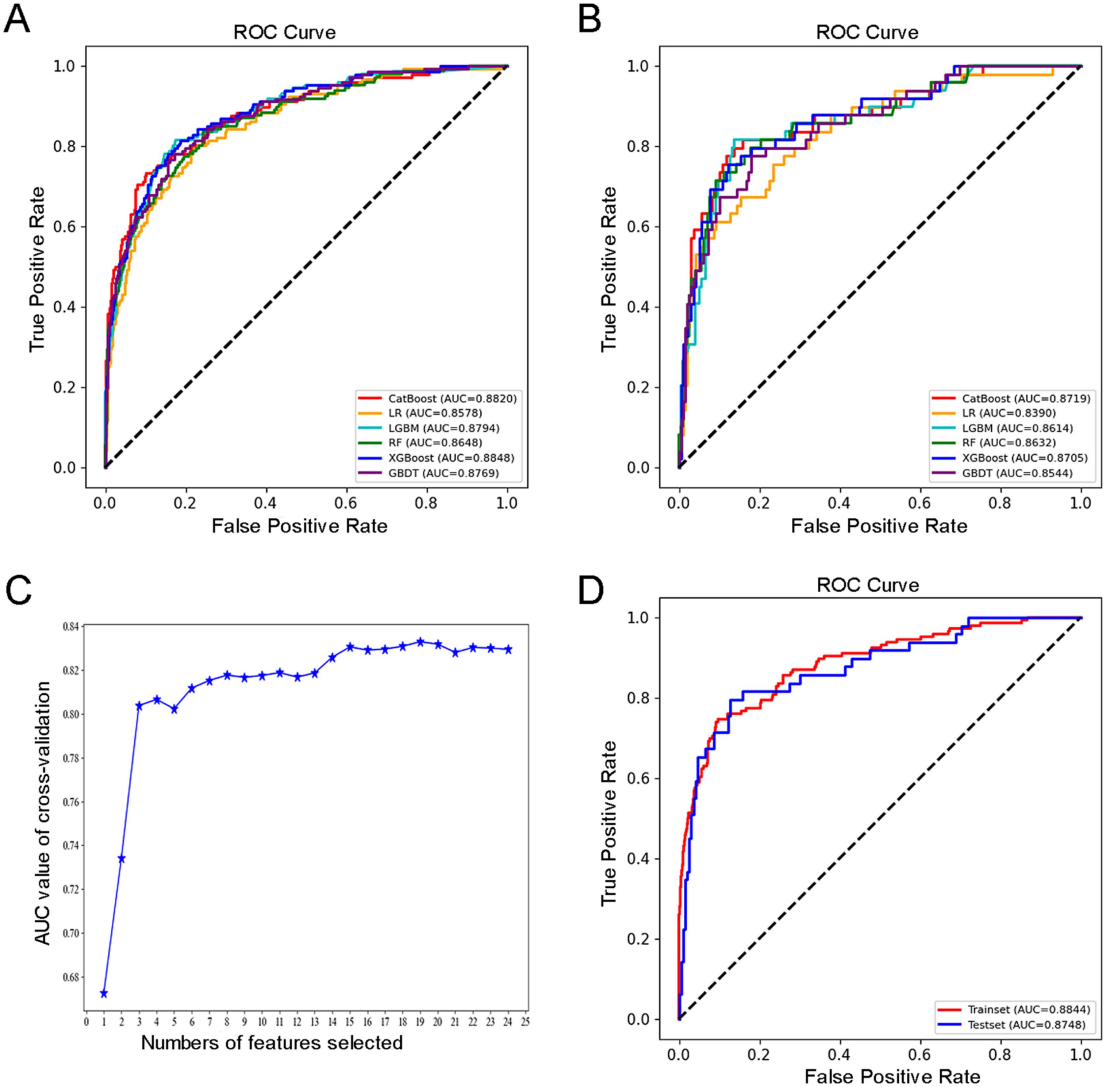
**(A)** ROC curves of six machine learning algorithms based on variables in the training dataset. **(B)** ROC curves of six machine learning algorithms based on variables in the testing dataset. **(C)** Recursive Feature Elimination (RFE) for feature screening. **(D)** ROC diagram of features modeled using Categorical Features Gradient Boosting (CatBoost).

For detailed results, refer to Table 3 and Figures 2A–C.

**Table 3:** Prediction performance of six machine learning algorithms.

Model	AUC training	AUC testing	Accuracy testing	Precision testing	Sensitivity testing	Specificity testing	F1 score testing
LR	0.8578	0.8390	0.8490	0.6250	0.6122	0.9082	0.6186
RF	0.8648	0.8632	0.8694	0.6604	0.7143	0.9082	0.6863
LGBM	0.8794	0.8614	0.8531	0.5970	0.8163	0.8622	0.6897
GBDT	0.8769	0.8544	0.8122	0.5205	0.7755	0.8214	0.6230
XGBoost	0.8848	0.8705	0.8531	0.6066	0.7551	0.8776	0.6727
CatBoost	0.8820	0.8719	0.8612	0.6230	0.7755	0.8827	0.6909

LR, logistic regression; RF, random forest; LGBM, light gradient boost machine; GBDT, gradient boosted decision tree; XGBoost, extreme gradient boosting; CatBoost, categorical features gradient boosting; AUC, area under the receiver operating characteristic (ROC) curve.

Using Recursive Feature Elimination (RFE) with the CatBoost algorithm, we identified and retained 19 key features: Age, Income, Comorbidity, Education Level, Living Alone Status, Use of Assistive Devices, History of Falls, Hearing Impairment, Vision Impairment, Alcohol Consumption, Sleep Disorder, Polypharmacy, ADL, BMI, Frailty Status, TUGT, 4-SBT, Five Times Sit-to-Stand Test (FTSST), and MMSE. The results of the modeling using the CatBoost algorithm are presented in Table 4 and Figures 2C–D.

**Table 4:** Modeling results using the CatBoost algorithm.

Model	AUC training	AUC testing	Accuracy testing	Precision testing	Sensitivity testing	Specificity testing	F1 score testing
CatBoost	0.8844	0.8748	0.8571	0.6094	0.7959	0.8724	0.6903

CatBoost, categorical features gradient boosting; AUC, area under the receiver operating characteristic (ROC) curve.

### Prediction results of the CatBoost model based on SHAP analysis

**Figure 3:**
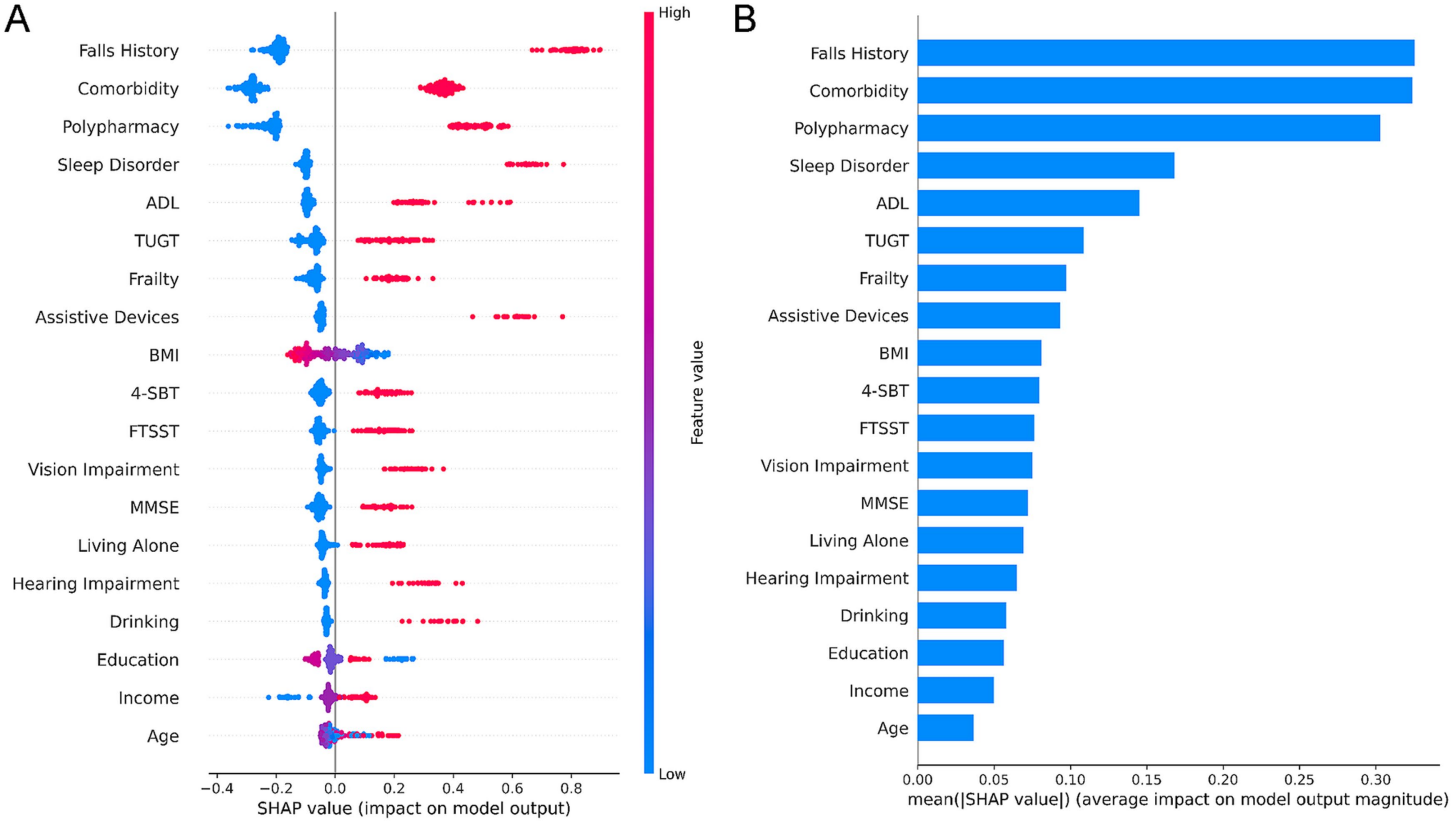
Interpretation of CatBoost via the SHAP method. **(A)** SHAP summary plot. **(B)** Feature importance ranking of the CatBoost model.

The two SHAP plots (Figure 3) illustrate the contribution of features to the CatBoost model across different dimensions. The scatter-type SHAP plot on the left organizes features by importance along the vertical axis, while the horizontal axis indicates the direction and magnitude of each feature’s influence on the model output—positive SHAP values increase the likelihood of fall risk prediction, whereas negative values decrease it. The color gradient (blue → red) reflects the feature’s actual value; for example, in the ‘Falls History’ feature, red dots represent individuals with a history of falls, which correspond to higher positive SHAP values. These densely clustered values intuitively demonstrate that a prior history of falls significantly increases fall risk. The bar-type SHAP plot on the right quantifies the average effect size of each feature in terms of ‘mean (SHAP value)’, further confirming that Falls History, Comorbidity, and Polypharmacy—due to their stronger influence—are the most critical factors in predicting fall risk. The average SHAP values for each feature in the final prediction model are presented in Figure 3.

## Discussion

The results of this study indicate that the fall incidence among community-dwelling older adults is 20%. Key risk factors for falls identified in this study include a history of falls, comorbidities, polypharmacy, sleep disturbances, ADL, TUGT, frailty status, and the use of assistive devices (21, 22). These findings are consistent with those reported in previous studies (11, 23). As the population continues to age, falls among older adults have emerged as a significant public health challenge (24). According to the 2020 census, the population of individuals aged 65 years and older in China reached 190.64 million, accounting for 13.5% of the total population. The incidence of falls increases with age, underscoring the importance of addressing this issue (3). Falls in older adults are multifactorial, influenced by both intrinsic and extrinsic factors. Extrinsic factors may involve physical environmental conditions, caregiving processes, and staffing levels (25), while intrinsic factors encompass patient-specific risks such as dizziness, weakness, and gait instability. Therefore, understanding the risk factors associated with falls among community-dwelling older adults is critical for the early identification of at-risk individuals and the development of effective prevention strategies.

In recent years, the increasing production of larger and more complex medical datasets, combined with advancements in artificial intelligence, has facilitated the widespread adoption of machine learning algorithms across various domains, including clinical practice. For instance, Marschollek et al. (26) performed data mining on assessments of hospitalized older patients to construct a classification decision tree for fall prediction. Their model achieved an overall accuracy of 66.0%, with a sensitivity of 55.4% and specificity of 67.1%. The positive predictive value was 15.0%, while the negative predictive value was 93.5%. This study underscores the potential of data-driven methodologies in identifying high-risk patients. Similarly, Ye et al. (27) employed an extreme gradient boosting algorithm to analyze electronic health records from 165,225 older patients, developing a 1-year fall prediction model with an AUC of 0.807. Notably, 50% of individuals classified as “high risk” experienced falls within the first 94 days of the subsequent year, highlighting the effectiveness of machine learning in long-term fall risk assessment. Liu et al. (28) investigated multiple machine learning algorithms for predicting fall risk at various stages during hospitalization, including admission, 24 h post-admission, peak clinical variables, and just prior to a fall event. Their results demonstrated that these models could enable continuous monitoring of fall risk throughout the hospital stay. Importantly, the ensemble classifier outperformed individual classifiers, emphasizing the benefits of integrating multiple models to enhance predictive accuracy.

However, the majority of existing research has predominantly focused on hospitalized patients, with relatively few studies addressing fall risk among community-dwelling older adults. Machine learning incorporates a diverse range of algorithms, each tailored to specific learning methods and applications. Consequently, it is crucial to evaluate the predictive performance of various algorithms to determine the most appropriate model for a given context. In this study, we developed a fall risk prediction model for community-dwelling older adults using machine learning techniques, including LR, RF, LGBM, CatBoost, GBDT, and XGBoost. Among these, the model based on the CatBoost algorithm exhibited the highest AUC and accuracy. Falls among community-dwelling older adults constitute a significant safety management challenge that requires the precise identification of individuals at high risk.

In terms of overall model performance, CatBoost demonstrates a clear advantage in the task of fall risk prediction: it achieves the highest AUC (0.8719) and F1 score (0.6909) on the test set, while maintaining balanced sensitivity (0.7755) and specificity (0.8827). This indicates that CatBoost not only identifies individuals at high risk of falls with greater accuracy, but also maintains consistent discrimination between high-risk and non-high-risk populations. The ensemble learning architecture of CatBoost enables it to effectively capture complex interactions within heterogeneous healthcare data, resulting in superior generalization performance on the test set while preserving strong training fit (AUC 0.8820). In contrast, although XGBoost performs comparably to CatBoost in terms of test set AUC (0.8705), it exhibits slightly lower sensitivity (0.7551) and a reduced F1 score (0.6727), indicating diminished accuracy in identifying high-risk individuals. While the F1 scores of Random Forest (RF) and LightGBM (LGBM) are close to that of CatBoost, both models show inferior performance in terms of AUC and specificity. Notably, LGBM demonstrates a significantly lower precision value (0.5970), which may lead to an increased number of false-positive predictions. Overall, CatBoost’s consistently strong and balanced performance across all key evaluation metrics establishes it as the most suitable model for fall risk prediction in this study. Therefore, model selection should prioritize sensitivity while maintaining reliability. Based on these criteria, we determined that CatBoost algorithm was the optimal model for our research.

Compared to traditional statistical methods, machine learning approaches offer greater flexibility in handling multiple covariates, capturing non-linear relationships, and improving classification performance without the assumption of linearity. Furthermore, our use of SHAP values enhances model interpretability, enabling a more transparent understanding of risk factors at both population and individual levels. These advantages highlight the significant practical potential of machine learning in developing fall risk prediction models for community-dwelling older adults.

This study identifies several common factors influencing fall occurrence among older adults in the community. Specifically, a history of previous falls was found to be a critical risk factor for future falls, which aligns with findings from prior studies (29). Experiencing a fall may trigger fear of falling again, leading older adults to restrict daily activities and physical functioning, impair postural control responsiveness, and ultimately increase the likelihood of recurrent falls (12). Therefore, it is recommended that community healthcare providers incorporate fall history into initial screenings for fall risk assessment. Conducting an annual evaluation of whether an individual has experienced a fall within the past 12 months can help efficiently identify those at high risk.

Additionally, this study confirms that co-morbidities are significant contributors to fall risk among community-dwelling older adults, consistent with previous research (1). As the number of chronic conditions increases, their combined or synergistic effects can lead to greater disease burden, reduced functional capacity, impaired coordination and reaction time, diminished balance, and increased fall susceptibility (30).

Moreover, polypharmacy was associated with a higher incidence of falls among community-dwelling older adults, corroborating findings by González et al. (31). This is primarily due to age-related declines in metabolic capacity, making older adults more susceptible to pharmacokinetic and pharmacodynamic changes following drug administration. These physiological alterations can predispose individuals to falls (32). Secondly, the concurrent use of multiple medications involves complex mechanisms such as dysfunction, adverse drug–drug interactions, and negative physiological responses, which may act synergistically or antagonistically to produce harmful health outcomes, impair body control, and elevate fall risk (33).

This approach offers a practical solution for developing a generalizable and accurate fall risk prediction model suitable for settings without access to an EMR system. A wide range of easily obtainable fall risk predictors were collected through assessments conducted in community primary care settings, without requiring complex instrumentation, thereby enhancing the clinical applicability of the model. Additionally, we ranked the importance of fall risk predictors, allowing caregivers to optimize their time and resources by implementing targeted interventions for individuals at high risk of falls, thus improving the effectiveness of fall prevention strategies. Furthermore, family members of community-dwelling older adults should be more attentive to this population and adopt tailored approaches to prevent and address falls. Preventive measures should include home safety assessments and modifications—such as the installation of handrails, improved lighting, and elimination of tripping hazards—targeting individuals at high risk due to co-morbidities and multiple medication use. Early preventive actions should also be emphasized, along with safety education and awareness initiatives aimed at improving the overall safety of older adults in the community.

The model constructed in this study could serve as a screening tool to quickly identify older adults at high risk of falls in the community, and it provides a scientific basis for community medical staff and family members to assess fall risks and enhances their awareness of early warning for older adults fall risks. Key variables identified by the SHAP model (such as a history of previous falls, multiple comorbidities, multiple medications, etc.) can be utilized to develop targeted intervention plans and health education, thereby implementing precision fall prevention interventions and reducing the incidence of falls among community-dwelling older adults.

This study developed a fall risk prediction model for community-dwelling older adults using machine learning algorithms. The model’s performance was evaluated using six metrics, including the AUC, precision, and accuracy, which helps to minimize bias that may arise from relying on a single evaluation metric However, several limitations should be acknowledged: (1) Participants in this study may have been subject to reporting bias, as self-reported data may not always accurately reflect their actual health status. Underreporting or over-reporting of symptoms could have occurred due to social desirability bias or recall bias; (2) the sample was drawn from a single geographic region, which may have introduced selection bias. Due to the lack of external validation, future research should prioritize multi-center external validation to enhance the model’s predictive performance; (3) given that the participants were community-dwelling older adults, the absence of physiological and biochemical data limits the applicability and generalizability of the model; (4) this study only utilized baseline predictive values and did not include a prospective observational design to further validate these risk factors through the incorporation of dynamic, process-related variables into the predictive model.

## Conclusion

Falls among older adults are a globally recognized public health concern, posing substantial risks to both individuals and their families. Accurately predicting fall risk is essential for the timely identification of high-risk populations and the development of effective intervention strategies. This study utilized feature selection and optimization techniques, demonstrating that the final prediction model based on the CatBoost algorithm, incorporating 19 variables (such as history of falls, frailty status, and polypharmacy), exhibits robust predictive performance for community-dwelling older adults. Furthermore, SHAP analysis provides deeper insights into how the selected variables influence fall risk, thereby complementing the prediction results. However, given the diversity of machine learning algorithms, each with its own strengths and limitations, further research is warranted to determine the most appropriate algorithm for clinical applications and specific population characteristics. Future research will focus on refining algorithmic structures and parameters to enhance the effectiveness and generalizability of predictive models.

## Data Availability

The raw data supporting the conclusions of this article will be made available by the authors, without undue reservation.
